# Exploring if day and time of admission is associated with average length of stay among inpatients from a tertiary hospital in Singapore: an analytic study based on routine admission data

**DOI:** 10.1186/1472-6963-6-6

**Published:** 2006-01-22

**Authors:** Arul Earnest, Mark IC Chen, Eillyne Seow

**Affiliations:** 1 Department of Clinical Epidemiology, Tan Tock Seng Hospital, Singapore; 2 Emergency Department, Tan Tock Seng Hospital, Singapore

## Abstract

**Background:**

It has been postulated that patients admitted on weekends or after office hours may experience delays in clinical management and consequently have longer length of stay (LOS). We investigated if day and time of admission is associated with LOS in Tan Tock Seng Hospital (TTSH), a 1,400 bed acute care tertiary hospital serving the central and northern regions of Singapore.

**Methods:**

This was a historical cohort study based on all admissions from TTSH from 1^st ^September 2003 to 31^st ^August 2004. Data was extracted from routinely available computerized hospital information systems for analysis by episode of care. LOS for each episode of care was log-transformed before analysis, and a multivariate linear regression model was used to study if sex, age group, type of admission, admission source, day of week admitted, admission on a public holiday or eve of public holiday, admission on a weekend and admission time were associated with an increased LOS.

**Results:**

In the multivariate analysis, sex, age group, type of admission, source of admission, admission on the eve of public holiday and weekends and time of day admitted were independently and significantly associated with LOS. Patients admitted on Friday, Saturday or Sunday stayed on average 0.3 days longer than those admitted on weekdays, after adjusting for potential confounders; those admitted on the eve of public holidays, and those admitted in the afternoons and after office hours also had a longer LOS (differences of 0.71, 1.14 and 0.65 days respectively).

**Conclusion:**

Cases admitted over a weekend, eve of holiday, in the afternoons, and after office hours, do have an increased LOS. Further research is needed to identify processes contributing to the above phenomenon.

## Background

Optimising of length of stay is one approach to improving hospital performance, particular under funding systems that quantify outputs by the episode of care, as unnecessary inpatient bed-days constitute a significant component of per-episode costs. Casemix funding of inpatient services was introduced to Singapore in October 1999,[1] and it was based on the Australian National Diagnoses Related Group or AN-DRG system. The DRG system, which funds acute public sector hospitals such as Tan Tock Seng Hospital (TTSH), has given additional impetus towards finding factors which may be responsible for prolonging length of stay (LOS).

Contribution to inappropriate LOS may include patient, disease-related, as well as institutional and organizational factors. While the actual factors differ for various individual DRGs, it is foreseeable that certain institutional factors may cut across multiple disciplines and disease groups in a systemic fashion. From a hospital administrator's perspective, it would be useful to look at the overall group of patients and identify such institutional and organizational factors as well, as these could be amenable to the reorganization of service delivery at the institutional level.

Clinicians and other front-line staff have often suspected that the reduction of service capacity over the weekend delays the investigation of new cases, hence prolonging the length of stay. This effect would be most pronounced in cases admitted just before, or on the weekend itself, since the initial workup for the admission episode would be delayed for these patients. There is some published evidence that day of admission and access to health care services can affect LOS. A study in Germany on stroke patients showed that LOS is associated with the day of the week the patient was admitted[2] Another study based on patients with acute exacerbation of chronic obstructive pulmonary disease (COPD) in Spain also reported that weekend admission was associated with a prolonged length of stay, which they defined as more than 3 days[3] These studies have looked at patients within a specific disease group, and to the best of our knowledge, no studies have attempted to explore the association of time and day of admission with LOS at the hospital level. Information on associations at the hospital level could prove useful in generating hypothesis and identifying key areas for further investigation and improvement. For example, the persistence of the effect across different specialities may point to a common dependency on specific bottleneck investigations or procedures that are not available during certain periods; alternatively, if the analysis showed that the effect was restricted to specific departments, then the solution would be to optimise resources within those specific disciplines.

We hence set out to examine if day and time of admission is associated with length of stay among TTSH inpatients, while adjusting for possible differences in the timing-related profile of admission episodes.

## Methods

### Study design and setting

This was a historical cohort study design on patients admitted to TTSH, a 1,400 bed acute care tertiary hospital serving the central and northern regions of Singapore. Admissions from TTSH from 1^st ^September 2003 to 31^st ^August 2004 were included in the analysis. Those who died were excluded from the analysis (less than 5% of all admissions). In addition, we excluded day surgery cases and other inpatient stays with less than one day.

### Data extraction

We used only data from routine administrative sources that are part of the Hospital Management Information Systems. Data on admissions and discharges was extracted with the assistance of the information technology department, and organized by the episode of care. Further data management was performed in MS Access 2000 to process the additional covariates of interest with regards to day of week, relation in time to public holidays, and the timing of the admission episodes.

### Analysis

We then used the multivariate linear regression model to study the factors affecting length of stay,[4] with each admission episode as the primary unit of analysis. LOS was transformed and analysed on the natural logarithmic scale, as the residuals from the model were not normally distributed, and the variable was highly skewed. For the final model, we back-transformed the coefficients and calculated the difference in the average LOS between sub-groups within each factor.

Among the covariates studied were sex, age group, type of admission, admission source, day of week admitted, admission on a public holiday or eve of public holiday, admission on a weekend (defined as Friday, Saturday or Sunday) and admission time (defined as 0800 to 1229 hrs, 1230 to 1659 hrs and 1700 to 0759 hrs). For the final model, we checked for normality and heterogeneity of the residuals and used the variance inflation factor (VIF) to examine multi-collinearity between explanatory variables[5] Data analysis was performed in Stata (V7.0), and all tests were conducted at the 5% level of significance.

## Results

There were 45395 episodes of care included in the analysis for the study period. Table 1 summarizes the sample characteristics. Slightly more than half were male, and the median age was 58 years. The majority were emergency admissions. In terms of day of admission, the daily admissions were similar across the days, except for weekends, when there was a noticeable decline in the number of admissions. Slightly more than a third of all cases (37%) were admitted over the weekend, and a small percentage on the 11 public holidays and eves of public holidays. In terms of time of day admitted, slightly more than half were admitted during the two office hour periods (0800 to 1229 and 1230 to 1659 hrs), with the rest being admitted after office hours (1700 to 0759 hrs the next day).

In the univariate analysis, we found that sex, age group, type of admission, source of admission, class on admission, day of week admitted, admission on a public holiday, eve of public holiday and weekends and also time of day admitted were significantly associated with length of stay (Table 2).

However, in the multivariate analysis, only sex, age group, type of admission, source of admission, admission on the eve of public holiday, admission on weekends, and time of day admitted were independently associated with LOS (Table 3).

In terms of demographics, males generally stayed around 0.3 days lesser than females, and those who were older stayed longer. Those who were admitted on weekends stayed on average 0.3 days longer than those admitted on weekdays, after adjusting for the other significant covariates shown in Table 3, and this difference was found to be statistically significant (p < 0.001). We also found that those admitted in the afternoons and after office hours stayed longer (1.1 and 0.7 days respectively) than those admitted in the mornings.

Elective admissions stayed around 2 days lesser than emergency admissions, and this was also found to be statistically significant (p < 0.001). For source of admission, direct ward-to-ward admissions stayed the longest. However, this association was likely confounded through an artifact of administrative classification, as most of these were cases admitted from another service within the hospital to the neurology service (NLD and NSD), which functioned under an independent management within TTSH; the observed increased LOS in ward-to-ward admissions hence reflects the complicated nature of this special group of admission episodes. There was a change in direction of effect for those admitted from SOC after adjusting for other covariates. In the univariate analysis, we found that SOC admissions stayed lesser than emergency admissions, whereas in the multivariate model, SOC admissions stayed longer. This could be due to the suppressor effect of other confounders, in particular the type of admission, which is partially correlated with the source of admission.

In addition to the overall model, we did further sub-group analysis, by studying the difference in LOS by the top 5 admitting disciplines by volume. As we can see in Table 4, the greatest effect was seen in patients admitted under General Surgery, where the difference in LOS between weekend and weekday admissions was found to be 0.57 days, after adjusting for potential confounders, and this was found to be statistically significant (p < 0.001). This was followed by Neurology (0.55 days) and Respiratory Medicine (0.50 days). We did not find any significant difference between weekend and weekday admissions for General Medicine and Orthopaedics.

We also performed a stratified analysis for 28,739 admissions for which DRG codes were available at the time of data extraction (mostly in admissions from the earlier two thirds of the dataset), focussing on the difference in LOS for weekend and weekday admissions. The results for the top three DRGs by volume for the above 5 disciplines are presented in Table 5. Longer LOS for weekend admissions was not consistently observed across the top 3 DRGs in the 5 disciplines, but appears predominantly in DRGs for short-stay admissions without co-morbidities and complications. The most marked and significant effects were for DRG 330 in General Surgery, and DRG 38 for Neurology.

To determine if the effects on LOS could be explained by differences in disease severity, we also stratified the analysis into direct admissions to intensive care / high-dependency units (ICU/HD), and admissions to the general ward (GW). The adjusted difference in average length of stay among 1517 direct admissions to ICU/HD for weekends versus weekdays was 0.7 days; this was not found to be significant (p = 0.189). However, the difference in ALOS between weekend and weekday admissions for those who admitted to GW was 0.3 days, and this was statistically significant (p < 0.001).

In terms of model diagnostics, the residuals were fairly normally distributed, with a homogeneous variance. The variance inflation factors for all the variables were below 3, indicating the absence of multi-colinearity.

## Discussion

The results from this study confirmed the primary hypothesis that patients admitted on Friday, Saturday or Sunday stayed on average 0.31 days longer than those admitted on weekdays, after adjusting for potential confounders. However, we found that the timing of admission had an even greater effect on length of stay than initially suspected, in that those admitted on the eve of public holidays, and those admitted in the afternoons and after office hours also had a longer LOS (differences of 0.71, 1.14 and 0.65 days respectively). Of particular interest is the fact that the greatest difference in LOS was for those admitted in the afternoons – we could hypothesize that these admission episodes are missing about a day's worth of clinical work-up and management by being admitted past the optimal timing for ordering tests and procedures, which would usually be in the mornings. The above observations are hence consistent with the suspicion of clinical and front-line staff that decreased service levels available after office hours, on weekends, and during public holidays prolong LOS through delays in obtaining the necessary initial work-up for newly admitted cases. Our conclusion is similar to the study done by Iglesia and colleagues [3], who also found that weekend admission was an independent prognostic factor in determining the length of stays in patients with COPD (dichotomized as more than 3 days versus less than or equal to 3 days). On the other hand, our study results show that this phenomenon is applicable to a wider patient pool (not just COPD patients), and we have also been able to quantify the estimated difference in LOS between those admitted on weekends and weekdays, which is useful for further cost-effectiveness studies.

While the effect size reported in our study may appear small, the potential cost savings of successful programs to reduce the length of stay from admission timing should not be underestimated. For instance, if introduction of a staggered work-week could effectively reduce the LOS for the 16,994 admissions on weekends to that observed for admissions on weekdays, we could potentially save 5,207 bed-days, which would then amount to around about S$1.5 million per year in cost-savings in terms of ward-costs alone, assuming an average of S$300 per day (based on internal cost data from the TTSH Finance Department). Alternatively, a shift system allowing services to be maintained at the optimal level across a 24-hour day could have an even greater impact, as it would save about 17,600 and 13,900 bed-days in those admitted in the afternoons and after office hours respectively. This may be more difficult to implement than a staggered workweek, but could amount to greater cost-savings in excess of S$9 million per year. Although the premium for maintaining the services around the clock has to be factored into cost-benefit calculations, the above may still be underestimates of the full impact, because appropriate increments in service levels at critical timings would not just reduce LOS in the newly admitted cases, but also in other admission episodes, such as those admitted mid-week but whose stay overlaps into the weekend, for which delays may occur due to the timing-related changes in service levels.

With regards to the speciality specific analysis, it was interesting to note the differences observed between General Surgery and Orthopaedics, which are both surgical disciplines admitting acute cases, as well as between Respiratory Medicine and General Medicine, the key admitting disciplines for acute medical cases. While weekend admissions were associated with prolonged LOS for General Surgery, the effect is not borne out for Orthopaedics. One hypothesis could be that admission on a weekend delays key procedures for General Surgery patients, but not for Orthopaedics. Analysing the dates and times of operations in the two disciplines against day of admission could test such a hypothesis. Information on the type and timings of operations performed can also be extracted from routinely available administrative data and linked to admission data. This could be attempted as a follow-up study. The difference in effect between Respiratory Medicine and General Medicine, however, is more difficult to explain. One approach is to compare LOS for similar DRGs across the two disciplines, while adjusting for the potential confounders identified. Such a comparison is possible in Table 5, where two out of the top three DRGs for each specialty are common to both (DRG 177 and DRG 170). There is some suggestion that the difference between weekend and weekday admissions for DRG 177 is greater in Respiratory Medicine than in General Medicine, but more detailed sub-analysis of the data for other DRGs would be necessary before conclusions can be drawn. However, this brief sub-analysis by DRGs does generate further hypotheses about possible causes for prolonged LOS in weekend admissions – it is noted that DRG 330 (Other Gastroscopy for Major Digestive Disease W/O CC) entails procedural intervention in the form of gastroscopy, and DRG 38 (Cerebrovascular Disorders Except TIA W/O CC) often necessitates referral to paramedical services such as speech and physical therapy. Such hypotheses can be followed up through qualitative methods to identify if service levels in any specific area may be responsible for the delays, but it would appear that the delays are more specific to disease groupings rather than to disciplines.

Based on the existing analysis, we hypothesize that the service levels concerned may be broadly categorized into two types. Firstly, there is decreased medical decision-making capability, both on weekends and after office-hours. For example, ward rounds are not performed on a 24-hour basis, and there is a reduction in the ratio of senior staff to patients on weekends. The other possible bottleneck in service levels would be in the availability of critical support and paramedical services, such as investigations, allied health inputs and inter-disciplinary referrals for key procedures. A recent paper by Bell and Redelmeier [6] provides strong evidence that part of the cause does lie in the wait for procedures, with patients admitted on Fridays and Saturdays having the longest delay from admission to procedure. Bell also found that the weekend effect was procedure-specific, and this may explain the variation we observe between specialties. In all, the evidence points towards amenable causes, and a sensible approach would hence be to look for actionable factors within the specific disciplines, so as to find cost-effective ways of enhancing decision-making capability and making critical services and procedures available on a weekend.

One limitation inherent in our study was the possibility that unmeasured confounders could have affected our results. Since the source of data for this report is primarily from administrative and routine information systems, clinical data that could help us make more detailed inferences was not available. In particular, there could be concerns that the severity of the cases remains an unmeasured confounder in our study. At least two studies have found that more severe cases tend to be admitted more frequently on weekends[7,8] However, both of these studies were based in intensive care units, whereas our study looks at all admissions, the vast majority of which are to general wards. It would be reasonable to assume that general ward admissions are less likely to be affected by fluctuations in severity by day of week, although we are unable to confirm this through the data available to us. However, we have attempted to deal with the issue of confounding by disease severity within the limitations of our dataset. Firstly, we performed a sub-analysis stratifying the data into direct admissions to ICU/HD and those initially admitted to the general ward, and found that the timing-related effects persisted at similar magnitudes in the admissions to general ward. Secondly, the sub-analysis by top DRGs (Table 5) suggests that the findings of timing-related effects are more applicable to short-stay admissions without co-morbidities and complications, and hence supports our contention that service levels rather disease severity are at work in causing this phenomenon. Lastly, we note that afternoon admissions have the longest LOS. While past studies have reported that weekend admissions may be more ill, afternoon admissions have never been noted have a greater disease severity in existing literature, and it would seem more plausible that the longer LOS in this group at least is the result of service delivery factors rather than illness severity.

## Conclusion

We were able to use routine administrative data to show that weekend admissions, as well as afternoon and after-office hour admissions, are associated with increased LOS at the hospital level. Because we used only routinely available administrative data, the study could be easily replicated in other settings. The information yielded in such analyses could confirm if the effect also exists in other hospitals, help to identify the specialities and disease groupings most affected, and provide the impetus for hospital administrators to invest further resources in identifying factors amenable to organizational changes. We postulate that the factors involve possible bottlenecks in service provision over weekends and after office hours. Further studies, possibly by major disciplines and disease conditions, are needed to identify the processes that constitute these critical bottlenecks.

## Competing interests

The author(s) declare that they have no competing interests.

## Authors' contributions

MIC conceived the study and contributed to the study design, analysis, interpretation and writing of the manuscript. AE contributed to the statistical analysis, interpretation and writing of the manuscript. ES contributed to the interpretation and writing of the manuscript.

## Pre-publication history

The pre-publication history for this paper can be accessed here:



## Figures and Tables

**Table 1:** Descriptive summary of study sample (n = 45395)

**Factor**	**n (%)**
**Sex**	
Female	20518 (45%)
Male	24877 (55%)
	
**Age group**	
Up to 34 years old	8349 (18%)
35 to 44 years old	5069 (11%)
45 to 54 years old	6626 (15%)
55 years and above	25351 (56%)
	
**Median age (IQR) in years**	58 (41–73)
	
**Admission type**	
Elective	4240 (9%)
Emergency	41155 (91%)
	
**Source of admission**	
Emergency Department	24394 (54%)
Specialist Outpatient Clinic	5098 (11%)
Ward	815 (2%)
Not Available	15088 (33%)
	
**Day of week admitted**	
Monday	7458 (16%)
Tuesday	7127 (16%)
Wednesday	6926 (15%)
Thursday	6890 (15%)
Friday	6442 (14%)
Saturday	5501 (12%)
Sunday	5051 (11%)
	
**Admission on**	
Public holiday	1137 (3%)
Eve of public holiday	1255 (3%)
Weekend (Fri-Sun)	16994 (37%)
	
**Admission time**	
0800 to 1229 hrs	8352 (18%)
1230 to 1659 hrs	15613 (34%)
1700 to 0759 hrs	21430 (47%)

**Table 2:** Univariate factors associated with length of stay

**Factor**	**Coefficient**	**95%CI**	**p-value**
**Sex [Female]**			
Male	-0.09	-0.11, -0.08	<0.001
			
**Age group [Up to 34 years]**			
35 to 44 years old	0.12	0.09, 0.15	<0.001
45 to 54 years old	0.24	0.21, 0.26	<0.001
55 years and above	0.59	0.57, 0.61	<0.001
			
**Admission type [Emergency]**			
Elective	-0.4	-0.43, -0.38	<0.001
			
**Source of admission [Emergency Department]**			
Specialist Outpatient Clinic	-0.17	-0.20, -0.15	<0.001
Ward	0.29	0.23, 0.35	<0.001
Not Available	0.09	0.07, 0.10	<0.001
			
**Day of week admitted [Monday]**			
Tuesday	0.05	0.02, 0.08	<0.001
Wednesday	0.01	-0.02, 0.03	0.605
Thursday	0.04	0.01, 0.06	0.012
Friday	0.11	0.08, 0.14	<0.001
Saturday	0.13	0.10, 0.16	<0.001
Sunday	0.03	0.00, 0.06	0.024
			
**Admission on:**			
Public holiday	0.08	0.03, 0.13	0.002
			
Eve of public holiday	0.12	0.07, 0.17	<0.001
			
Weekend (Fri-Sun)	0.07	0.06, 0.09	<0.001
			
**Admission time [0800–1229 hrs]**			
1230 to 1659 hrs	0.24	0.21, 0.26	<0.001
1700 to 0759 hrs	0.18	0.16, 0.20	<0.001

Note: Reference groups are indicated in parenthesis []

**Table 3:** Multivariate factors associated with length of stay

**Factor**	**Coefficient**	**95%CI**	**p-value**	**Diff in days**
**Sex [Female]**				
Male	-0.04	-0.05, -0.03	<0.001	-0.28
				
**Age group [Up to 34 years]**				
35 to 44 years old	0.12	0.09, 0.15	<0.001	0.51
45 to 54 years old	0.24	0.22, 0.27	<0.001	1.08
55 years and above	0.58	0.56, 0.60	<0.001	3.06
				
**Admission type [Emergency]**				
Elective	-0.43	-0.46, -0.40	<0.001	-2.35
				
**Source of admission [Emergency Department]**				
Specialist Outpatient Clinic	0.10	0.07, 0.13	<0.001	0.67
Ward	0.42	0.36, 0.47	<0.001	3.25
Not Available	0.13	0.11, 0.14	<0.001	0.86
				
**Admission on Eve of public holiday**	0.10	0.06, 0.15	<0.001	0.71
				
**Admission on Weekend (Fri-Sun)**	0.05	0.03, 0.06	<0.001	0.31
				
**Admission time [0800–1229 hrs]**				
1230 to 1659 hrs	0.18	0.16, 0.20	<0.001	1.14
1700 to 0759 hrs	0.10	0.08, 0.13	<0.001	0.65

Note: Reference groups are indicated in parenthesis []

**Table 4:** Difference in weekend and weekday length of stay in top 5 admitting disciplines (by volume)

		**Geometric mean**			
				
**Discipline**	**n**	**Weekday**	**Weekend**	Adjusted difference in days*	**p-value**
					
**General Medicine**	11220	5.1	5.3	0.13	0.209
					
**General Surgery**	7846	3.0	3.5	0.57	<0.001
					
**Ortho**	5636	4.3	4.3	-0.18	0.265
					
**Respiratory**	4150	4.4	4.9	0.50	0.002
					
**Neurology (NLD)**	3575	4.4	4.9	0.55	0.013

* note: difference for weekend versus weekday admission, adjusted for sex, age group, type and source of admission, admission time and admission on eve of public holiday

**Table 5:** Difference in weekend and weekday length of stay for the top 3 DRGs (by volume) of the top 5 admitting disciplines

	**n**	**Crude ALOS**	**Diff in ALOS (weekend – weekday)**	**p-value**
**General Medicine**				
DRG: 177 Chronic Obstructive Airways Disease	517	4.59	0.33	0.332
DRG: 170 Respiratory Infections/Inflamns Age>54 W CC	364	8.66	0.07	0.933
DRG: 889 Poisoning/Toxic Effects of Drugs Age<60 W/O CC	220	2.40	0.46	0.008
				
**General Surgery**				
DRG: 330 Other Gastroscopy for Major Digestive Disease W/O CC	259	2.73	0.87	<0.001
DRG: 318 Anal & Stomal Procedures W/O CC	214	1.78	0.17	0.176
DRG: 314 Appendectomy W/O Complicated Principal Diagnosis	213	2.66	0.21	0.187
				
**Orthopaedics**				
DRG: 455 Medical Back Problems Age<75 W/O CC	323	2.77	0.1	0.624
DRG: 420 Lower Extremity&Humerus Procs Exc Hip, Foot, Femur Age<60 W/O CC	199	3.60	-0.34	0.38
DRG: 432 Hand or Wrist Procedures Exc Major Joint	177	1.92	0.07	0.679
				
**Respiratory**				
DRG: 177 Chronic Obstructive Airways Disease	319	4.18	0.68	0.072
DRG: 170 Respiratory Infections/Inflamns Age>54 W CC	184	8.20	1.61	0.195
DRG: 171 Respiratory Infections/Inflamns (Age>54 W/O CC) or (Age<55 W CC)	127	5.33	-1.29	0.069
				
**Neurology**				
DRG: 37 Cerebrovascular Disorders Except TIA W CC	473	9.89	0.83	0.477
DRG: 38 Cerebrovascular Disorders Except TIA W/O CC	443	4.70	1.07	0.025
DRG: 47 Seizure Age<65 W/O CC	206	2.46	0.47	0.027

* note: difference for weekend versus weekday admission, adjusted for sex, age group, type and source of admission, admission time and admission on eve of public holiday
